# Hospital Finance

**Published:** 1923-01

**Authors:** Chas. E. S. Flemming


### CORRESPONDENCE.

[Correspondence on all subjects is invited, but we cannot be responsible for the opinions expressed by our correspondents, who must give name and address as a guarantee of good faith, but not necessarily for publication. Correspondents are reminded that conciseness greatly facilitates early insertion.]

### Hospital Finance.

*To the Editor of* THE HOSPITAL AND HEALTH REVIEW.

SIR,—The proposal to establish a central fund for provincial hospitals is evidence that it is beginning to be realised that no hospital can now expect to stand by itself, and that no district should consider itself responsible for the support of one hospital only. The area from which any hospital draws its patients can rarely be precisely defined, and while it derives its chief financial support from the district which it nominally serves, it opens its wards to patients from places from which it gets practically no financial assistance. The larger the hospital, the bigger the town to which it belongs, the more important its work, the more marked is the difference between contributions of money and of patients from distant quarters, the more unfair the distribution of the burden.

The inhabitants of any one district should realise that when they have paid the cost of the maintenance of their local hospital, they have not discharged the whole of their obligation to hospitals.

One central hospital in this part of England in 1920 treated 360 patients from a neighbouring county at a cost of £3,850, the hospital receiving in voluntary contributions from that county in that year the sum of £521.

Again, in the county of Wilts. in the year 1920 I found that 730 persons were treated in hospitals outside the county, at a cost to those hospitals of nearly £7,000, the contributions from the county to those hospitals amounting so far as could be ascertained to little over £1,200, and so, although the county maintained all its own hospitals, practically without outside help, at a cost of about £31,000, it cannot be said to have done properly its duty in the way of hospital support.

The work that is done in our hospitals in regard to the causation, the diagnosis, the treatment and the prevention of disease is of such importance to the well-being of the community that it alone would justify, one might almost say necessitate, the existence of these institutions. If we recognise that every hospital is but part of a common service, that none is either an isolated unit or the rival of another, we shall understand why the collecting of funds in any one district by or on behalf of only one hospital cannot be altogether satisfactory.

If the onus of maintaining hospitals is to be shifted from the individual institutions to the people, we shall have to find not only a different method of collecting the funds, but another method of distributing them.

In the first place, we have to find out what is the liability. This is not difficult; it is an easy matter to get at the total hospital expenditure of the country and divide it among the population. The figures would, of course, vary from year to year, but that is not a material objection, and if we know the liability per head we know the amount required for any given district. For instance, the total expenditure of the voluntary hospitals of England and Wales, including London, was in 1921 £5,254,386. This divided equally among the whole population would give a sum of 2s. 9d. per head. In that case a district with a population of 20,000 would have to provide annually a sum of £2,750.

The amount of the fund required for a district would be regulated not by the need of any one hospital, but by the requirements of the people.

One of the most powerful influences in obtaining money for hospitals is the local interest; if any scheme of the sort is to be successful this factor must be made use of, but at the same time the public will have to be educated to extend their interests beyond the local hospital. Whether these funds should be held by the local collecting committees or whether they should be pooled in part or in whole are questions which need not here be discussed. It would be necessary for the areas to be arranged by some central body such as the Local Voluntary Hospitals Committee acting in concert with the hospitals in that area.

A satisfactory distribution of these funds would entail important changes in the control of the revenue of the hospitals. When a patient was admitted to a hospital, wherever it might be, the whole of his cost while there would be paid from the fund of the district to which he belonged. Payments on behalf of patients are in many instances actually made now, but they do not by any means meet the whole cost of the individual case, and the amount has to be made up from several different funds; it would be much better that all these should be pooled in the hospital fund. How far income derived from endowments should be used for this purpose is a question that each hospital would have to settle for itself. It might be used for the provision of better equipment or of extra accommodation.

There are, of course, all sorts of matters of detail to be arranged and many difficulties and prejudices to be overcome. I have no doubt that they could all be met but do not wish to overload this letter with too much detail, and the main points to which I wish to draw attention are: the recognition of the facts that no hospital is an isolated unit, that no section of the population is dependent on one hospital alone, that every member of the population has an interest in and a duty to perform to the hospitals and that the only satisfactory way to manage the finances of a hospital is to charge to the account of each patient his cost to the institution.

Yours faithfully,

CHAS. E. S. FLEMMING,

M.R.C.S., L.R.C.P.

